# Plant mediated fabrication of silver nanoparticles, process optimization, and impact on tomato plant

**DOI:** 10.1038/s41598-023-45038-x

**Published:** 2023-10-23

**Authors:** Madeeha Ansari, Shakil Ahmed, Asim Abbasi, Muhammad Tajammal Khan, Mishal Subhan, Najat A. Bukhari, Ashraf Atef Hatamleh, Nader R. Abdelsalam

**Affiliations:** 1 Institute of Botany, University of the Punjab, Lahore, 54590 Pakistan; 2 Department of Environmental Sciences, Kohsar University Murree, Murree, 47150 Pakistan; 3 School of Plant Sciences, University of Arizona, Tucson, AZ 85721 USA; 4 Department of Botany, Division of Science and Technology, University of Education, Lahore, 54770 Pakistan; 5 Department of Microbiology and Molecular Genetics, The Women University Multan, Multan, 66000 Pakistan; 6 Department of Botany and Microbiology, College of Science, King Saud University, P.O. Box 2455, Riyadh, 11451 Saudi Arabia; 7 Agricultural Botany Department, Faculty of Agriculture (Saba Basha), Alexandria University, Alexandria, 21531 Egypt

**Keywords:** Microbiology, Plant sciences

## Abstract

Nanotechnology is one of the fastest-growing markets, but developing eco-friendly products, their maximum production, stability, and higher yield is a challenge. In this study, silver nanoparticles were synthesized using an easily available resource, leaves extract of the Neem (*Azadirachta indica*) plant, as a reducing and capping agent, determined their effect on germination and growth of tomato plants. The maximum production of silver nanoparticles was noted at 70 °C after 3 h of reaction time while treating the 10 ml leaf extract of Neem plant with 10 ml of 1 mM silver nitrate. The impact of the extract preparation method and solvent type on the plant mediated fabrication of silver nanoparticles was also investigated. The UV-spectrophotometric analysis confirmed the synthesis of silver nanoparticles and showed an absorption spectrum within Δ420–440 nm range. The size of the fabricated silver nanoparticles was 22–30 nm. The functional groups such as ethylene, amide, carbonyl, methoxy, alcohol, and phenol attached to stabilize the nanoparticles were observed using the FTIR technique. SEM, EDX, and XRD analyses were performed to study the physiochemical characteristics of synthesized nanoparticles. Silver nanoparticles increased the germination rate of tomato seeds up to 70% while decreasing the mean germination time compared to the control. Silver nanoparticles applied at varying concentrations significantly increased the shoot length (25 to 80%), root length (10 to 60%), and fresh biomass (10 to 80%) biomass of the tomato plant. The production of total chlorophyll, carotenoid, flavonoids, soluble sugar, and protein was significantly increased in tomato plants treated with 5 and 10 ppm silver nanoparticles compared to the control. Green synthesized silver nanoparticles are cost-effective and nontoxic and can be applied in agriculture, biomedical, and other fields.

## Introduction

Nanoscience is now a leading science, providing opportunities for fundamental and applied research across the board of cognitive sciences. The emergence of nanoscience and nanotechnology is driving a technological revolution on a worldwide scale^1^. Nanotechnology is usually applied to materials ranging from 1 to 100 nm. Nanomaterials differ from their bulk materials by a wide range of characteristics, including size, physical strength, chemical reactivity, electrical conductance, magnetism, and optical effects^2^.

Different physical, chemical, and biological methods have been developed to synthesize stable nanomaterials with controlled size and shape^3^. There is poor reproducibility due to the sensitivity of physical methods to process parameters, limited control over particle size and composition, difficulties in scaling up the synthesis process for commercial production, and generates hazardous waste during the process, which may require proper disposal^4^. Chemical Synthesis uses synthetic chemicals of high cost and generates hazardous chemicals and by-products generated during the synthesis process^5^. Biological synthesis has advantages compared to the chemical and physical processes in terms of cost, environmental friendliness, and ease of scaling up for large-scale synthesis^6^. The use of beneficial microorganisms^7^, such as cell cultures of bacteria, fungi, and plants, makes the biological technique environment-friendly, cost-effective, and safe^8^. Synthesis of nanoparticles using plants is preferred over other biological sources due to its simplicity. It is a single-step procedure that does not require the time-consuming process of maintaining cell culture and a sterile environment^9^. Plants are useful in manufacturing nanoparticles as they are abundant, readily available, easy to handle, a source of several metabolites, and rich in pharmacological constituents that act as reducing and capping agents in the synthesis of nanoparticles^10^. Metal nanoparticles are synthesized using various parts of plants, including roots, seeds, stems, latex, flowers, and buds^11^. Various plants and their parts, such as fruit extract of *Phyllanthus emblica* L.^12^, leaves of Fenugreek^13^, *Curcuma longa* L.^14^, and peel of *Citrus reticulata* Blanco^15^ have been reported for the formation of nanoparticles.

Various factors, including temperature, pH, reaction time, metal salt volume, and plant extract volume influence the green synthesis of nanoparticles. The interaction of these factors is critical in determining the shape and size of synthesized NPs^16^. The temperature plays a crucial role in the synthesis of silver nanoparticles. Typically, the reaction takes place at room temperature, which is a prolonged process, but increasing the temperature of the reaction mixture can speed it up. The temperature range for the reaction is mostly set between 30 and 100 °C. Increasing the reaction temperature decreases the rate of Ag+ ions, leading to the homogeneous nucleation of silver nuclei. The process enables the production of small-sized silver nanoparticles^17^. Studies have shown that higher temperatures in the reaction mixture 17 lead to a decrease in the rate of nanoparticle synthesis but an increase in stability. Furthermore, it has been observed that silver nanoparticles produced at higher temperatures tend to have smaller sizes^18^.

The reaction time is crucial in synthesizing nanoparticles, as it allows for proper interaction between the salt and the reducing complex components found in the testing extract^19^. The reaction time begins when the reactant is added to the beaker and continues until the reaction occurs^20^. Plants with higher concentrations of secondary metabolites or phytochemicals reduce salt more efficiently. Conversely, plants with fewer reduced compounds take longer to reduce salt. However, plants with fewer secondary metabolites still quickly produce nanoparticles. The reaction time is influenced by factors such as the acidity or basicity of the reaction mixture, temperature, the reducing power of the plant extract, light intensity, enzymes, and secondary metabolites present in the extract^21^.

The pH level significantly impacts biomolecules, altering their electrical charges and potentially hindering their ability to cap and stabilize. It, in turn, can impact the growth of nanoparticles^22^. When synthesizing nanoparticles, the pH level determines their properties and reaction kinetics. Different plant extracts contain various bioactive components, such as polyphenols, flavonoids, and proteins, which can act as reducing agents but exhibit different reactivity at different pH levels. Optimizing the pH conditions during the synthesis is crucial to maximize the reduction efficiency and control the size and shape of the resulting nanoparticles. Additionally, the pH level affects the stability and aggregation behaviour 18 of the synthesized nanoparticles. The surface charge of nanoparticles strongly depends on pH, which affects their colloidal stability and tendency to aggregate. By enhancing the electrostatic repulsion between nanoparticles at specific pH values, it is possible to prevent or minimize aggregation, which is important for achieving uniform and stable dispersions of silver nanoparticles^18^^,^^23^.

The size and shape of AgNPs are controlled by adjusting the concentration and ratio of plant extract and precursor used. Htwe^24^ used Imperata cylindrical plant extract as the reducing agent and ascorbic acid as the capping agent to synthesize AgNPs with quasi-spherical morphology. Their research showed that increasing the concentration of AgNO_3_ (from 0.5 to 0.9 mM) while keeping the extract and ascorbic acid concentration constant resulted in a larger particle size (32.7 to 39.9 nm). They also found that the absorption peak was narrower at 0.5 mM AgNO_3_, indicating a lower size distribution. Khan et al.^25^ used *Piper betle* L. leaf aqueous extract to synthesize AgNPs and tested various concentrations of AgNO_3_ (1, 2, 3, and 4 mM) and extract (1:2, 1:4, and 1:8 dilution ratio to the crude extract) to determine the most optimal conditions. Their results showed that 2 mM of AgNO3 and a 1:4 dilution ratio of the extract produced the best results, and increasing the AgNO3 concentration led to the formation of larger nanoparticles with a shift in the UV–vis peak to higher wavelengths. However, higher concentrations of the extract could result in instability and aggregation of AgNPs.

The volume of leaf extract used in the process significantly impacts the formation of nanoparticles, including the time it takes to form. Leaf extracts are crucial in reducing ions, and using an appropriate volume significantly improves nanoparticle 19 formation efficiency^21^. It is recommended to test in varying quantities of 1, 5, 10, 15, 20, and 100 mL to determine the impact of plant extract. However, it is best to use smaller volumes, such as 1, 2, 3, 4, or 5 mL, for nanoscale particle creation. The concentration of the leaf extract, containing phenols, polyphenols, polysaccharides, tannins, and anthocyanins, significantly affects the average size of the silver nanoparticles. The factor is crucial in reducing ions and producing stable nanoparticles at the nanoscale. Therefore, optimizing the concentration of the extract is crucial in nanoparticle synthesis^26^.

AgNPs have drawn a lot of interest due to their unique physicochemical and biochemical characteristics, and as a consequence, it is now considered to be one of the fundamental nanoparticles in nanotechnology. Silver nanoparticles are among the most promising nanomaterials and are being used for different purposes such as wound healing, radiotherapy, food packing, and water disinfection^16,27–30^ due to their antimicrobial^31^ and antiviral properties^32–34^. The application of AgNPs to enhance soil quality^35^, pesticide function, and plant growth could significantly benefit agriculture because current projections take into account the need for substantial enhancement in agricultural production for the coming thirty years^36^.

Several reports indicate that appropriate concentrations of AgNPs play an important role in enhancing seed germination^37^ and plant growth^38^, improving the amount of chlorophyll and the efficacy of photosynthesis^39^, and improving the effectiveness of using fertilizer and water^40^. The size, nature, and quantity of nanoparticles determine their impact on plants^36,41^. The plant species, its growing conditions, sail properties, and bioavailability of AgNPs in the soil also affect the activity of silver nanoparticles^42^. The silver nanoparticles’ impact on edible crop plants should be evaluated before commercializing the product in markets worldwide^43^. Applications of nanomaterials, primarily biological application, depends on their synthesis methods. As physically and chemically synthesized AgNPs also have been reported to show a negative impact on plant growth. AgNPs inhibit root growth, cause leaf damage, and result in oxidative stress, producing reactive oxygen species (ROS), which harms the overall health of the plant, especially when used in high concentrations. AgNPs also release toxic silver ions (Ag+) into the environment, which are taken up by plants and accumulate in their tissues. These negative effects interfere with the plant’s ability to take in nutrients, hinder photosynthesis, and impede overall plant growth.

*Solanum lycopersicum* L. is one of the most consumed vegetables worldwide. It is also a source of health-promoting biochemicals, including several carotenoids and phenolic compounds^44^. The current study reports the plant mediated fabrication of silver nanoparticles using the leaf extract of *Azadirachta indica* (Neem plant). The study aimed to optimize some important factors such as time, temperature, pH, volume of plant extract, concentration of reagent, method of extraction and solvent type to obtain the maximum yield of stable AgNPs. The synthesized nanoparticles were subjected to various techniques, including UV-spectrophotometry, FTIR, XRD, EDX, and SEM to study the characteristics of prepared AgNPs. It also determines their impact on seed germination and growth of Tomato plants under laboratory and greenhouse conditions.

## Results

### Green synthesis of silver nanoparticles

#### Effect of temperature, time, pH, reagent concentration, and plant extract volume

**Figure 1:**
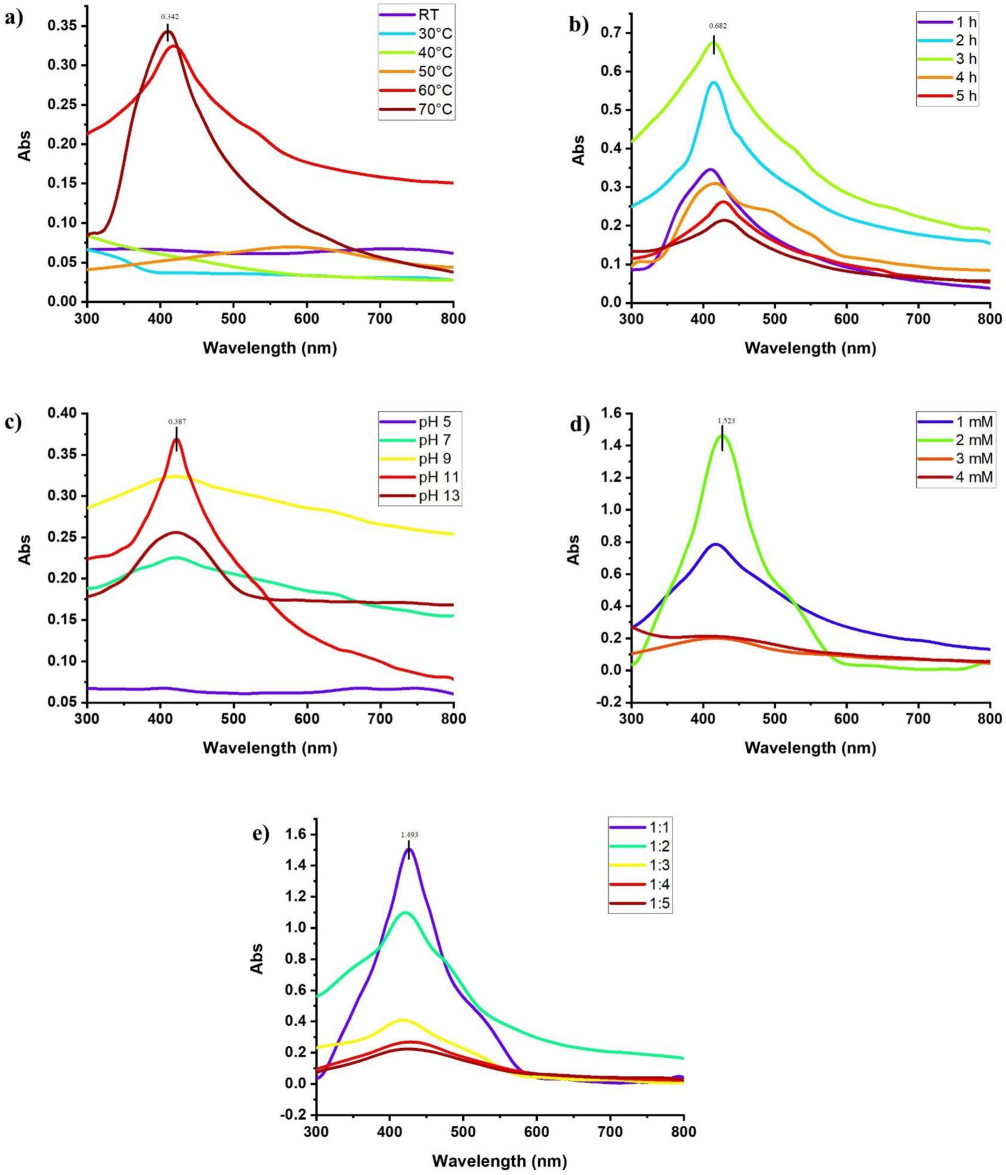
Effect of various (**a**) temperature after 1 h of reaction (**b**) time at 70 °C (**c**) pH (**d**) reagent concentrations (**e**) volume of plant extract on synthesis of AgNPs using leaf extract of Neem (*Azadirachta indica*) in distilled water using maceration method.

To determine the effect of temperature, the resulting solutions of leaf extracts were incubated at room temperature at 30, and 40 °C and did not show any SPR peak within the range of 400–500. The synthesis of AgNPs started upon increasing the temperature up to 50–70 °C. The solutions of S1E1 and S1E2 (details have been mentioned in the materials and methods) extracts kept at 70 °C gave more absorption rates of 0.342 and 0.279, respectively, than 60 °C as shown in Fig. 1a (maceration method) and 2a (boiling water method) and selected for further procedure. When both methanolic leaf extracts (S2E1 and S2E2) were reacted with AgNO_3_ at 50 °C, the synthesis of AgNPs was observed, and the UV spectrum gave a clear absorption peak. The increase in temperature increased the yield of AgNPs, and 70 °C was used for further procedure. The solutions of S3E1, S4E1, and S4E2 extracts also gave maximum production at 70 °C, while S3E2 exhibited a maximum absorption rate of 0.278 at 60 °C, further increase in temperature (70 °C) triggered the agglomeration in the reaction mixture and a decrease in absorption rate, therefore 60 °C was selected for further experiment.

**Figure 2:**
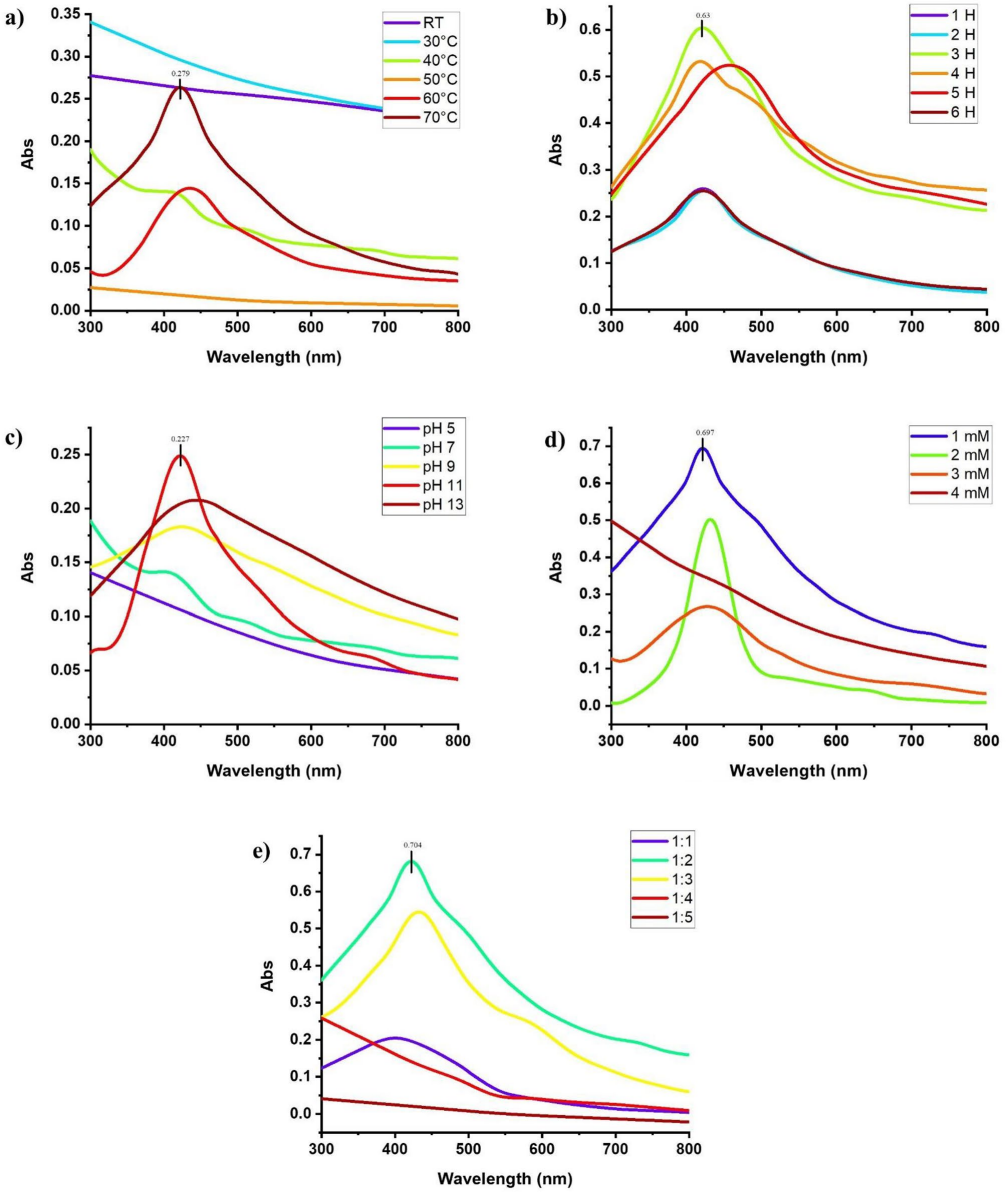
Effect of various (**a**) temperature after 1 h of reaction (**b**) time at 70 °C (**c**) pH (**d**) reagent concentrations (**e**) volume of plant extract on synthesis of AgNPs using leaf extract of (*Azadirachta indica*) in distilled water using boiling method.

To determine the effect of time, the leaf extract of each solvent was mixed with 1 mM silver nitrate solution in different vials and kept in an incubator at the selected temperature. The minimum absorption rate was noted after 1 h of reaction in the case of each solvent. The absorption rate of resulting solutions of S1E1 was more after 3 h (0.682) than 1 (0.342), 2 (0.589), 4 (0.325), 5 (0.273), and 6 h (0.225). A sharp and clear characteristic peak was observed within the range of 416–422 nm. The absorption rate of resulting solutions of S1E2 extract increased by increasing the time to 4 h (0.63) and then decreased. But 3 h time was selected with an absorption rate of 0.556 because the peak was shifted to a higher wavelength at 4 h, suggesting the production of larger AgNPs. Similarly, the methanolic extracts (S2E1 and S2E2) showed a maximum absorption rate after 3 h of reaction and minimum absorption at 6 h. The solutions of S3E and S3E2 extracts gave maximum production of AgNPs after 2 h (0.524 and 0.355, respectively) of reaction as the solutions exhibited higher absorption. In the case of S4E1 absorption rate was also increased by increasing the timing of the reaction up to 2 h but started to decrease after 3–6 h of reaction. In contrast, the resulting solution of S4E2 gave the maximum production of AgNPs after 4 h [Fig. 1b (maceration method) and Fig. 2b (boiling method)].

To determine the effect of pH, the leaf extracts reacted with silver nitrate solution in an incubator, making the pH of solution 5, 7, 9, 11, and 13. No color change was observed at pH 5 and 7, and the UV spectrum showed no characteristic peak. The aggregation of molecules in the mixture could be seen clearly. The production of AgNPs started with an increase in the pH of the solution up to pH 13. The solution kept at pH 13 gave a broad peak indicating the synthesis of bigger AgNPs. The absorption rates of 0.387, 0.361, 0.227, 0.328, 0.376, and 0.283 were given at characteristic peak within 420–435 nm by S1E1, S1E2, S2E1, S3E1, S4E1, S4E2, respectively. The maximum absorption rate (0.237) was shown when the solution mixture of S2E2 and silver nitrate was kept at pH 9 instead of 11. While in the case of S3E2, pH 13 gave the maximum absorption rate of 0.586 and a sharp characteristic peak [Fig. 1c (maceration method) and Fig. 2c (boiling method)].

To determine the effect of various reagent concentrations i.e., 1, 2, 3, and 4 mM of AgNO_3,_ were reacted with considered leaf extracts of the Neem plant. The leaf extract (S1E1), when treated with 1 mM and 2 mM silver nitrate solution, exhibited a color change from yellow to dark brown. The SPR peak was observed at 424 and 426 nm wavelengths, respectively. The peak was shifted to 412 nm at 3 mM concentration of silver nitrate, but the reaction mixture changed slightly in color. The maximum absorption rate (0.697) was presented when 1 mM solution of silver nitrate was reacted with S1E2 to synthesize nanoparticles. The leaf extract (S2E2), when treated with 1 mM and 2 mM silver nitrate solution, exhibited the characteristic peak at 432 and 420 nm wavelengths, respectively. A more absorption rate of 0.812 was shown in the 2 mM solution than in 1 mM. The maximum AgNPs were produced when 1 mM silver nitrate was reacted with S3E1. UV–vis spectrum displayed a characteristic peak at 424 and maximum absorbance (1.162). The leaf extracts S3E2 exhibited maximum absorption in a 2 mM reagent solution. The leaf extract of the Neem plant S4E1 and S4E2, when treated with 1 mM and 2 mM silver nitrate solution, exhibited a color change, and the characteristic peak was observed within 420–430 nm. The absorption rate again decreased at 3 mM concentration of AgNO_3,_ the color change was also slight, and aggregation occurred due to a higher concentration of the reagent in the mixture. The reaction mixture’s color didn’t alter, and the expected peak still appeared when 4 mM concentration was used, indicating no synthesis of AgNPs due to higher concentration of salt as compared to the presence of biomolecules in the extract [Fig. 1d (maceration method) and Fig. 2d (boiling method)].

To determine the effect of plant extract volume, the silver nitrate solution was added in 10, 20, 30, 40, and 50 mL of each leaf extract in different vials and kept in an incubator. The lowest absorbance and slight color change were shown when 10 mL reagent was reacted with 40 and 50 mL of each plant extract, and a characteristic peak was observed in the UV spectrum. When the volume of plant extract was decreased, the absorbance was increased, suggesting the reduction of silver into silver nanoparticles. The maximum absorption rate was shown when the reagent was reacted with plant extracts S1E1 and S1E2, respectively, in a 1:2 ratio. The maximum absorbance was 0.974 (S2E1) and 0.792 (S2E2) at 420 nm, shown with a clear SPR peak when the silver nitrate and plant ratio was 1:3. The color changed from brown to dark brown. The plant extracts S3E1, S3E2, S4E1, and S4E2 demonstrated the same pattern [Fig. 1e (maceration method) and Fig. 2e (boiling method)].

#### Effect of solvent type

**Figure 3:**
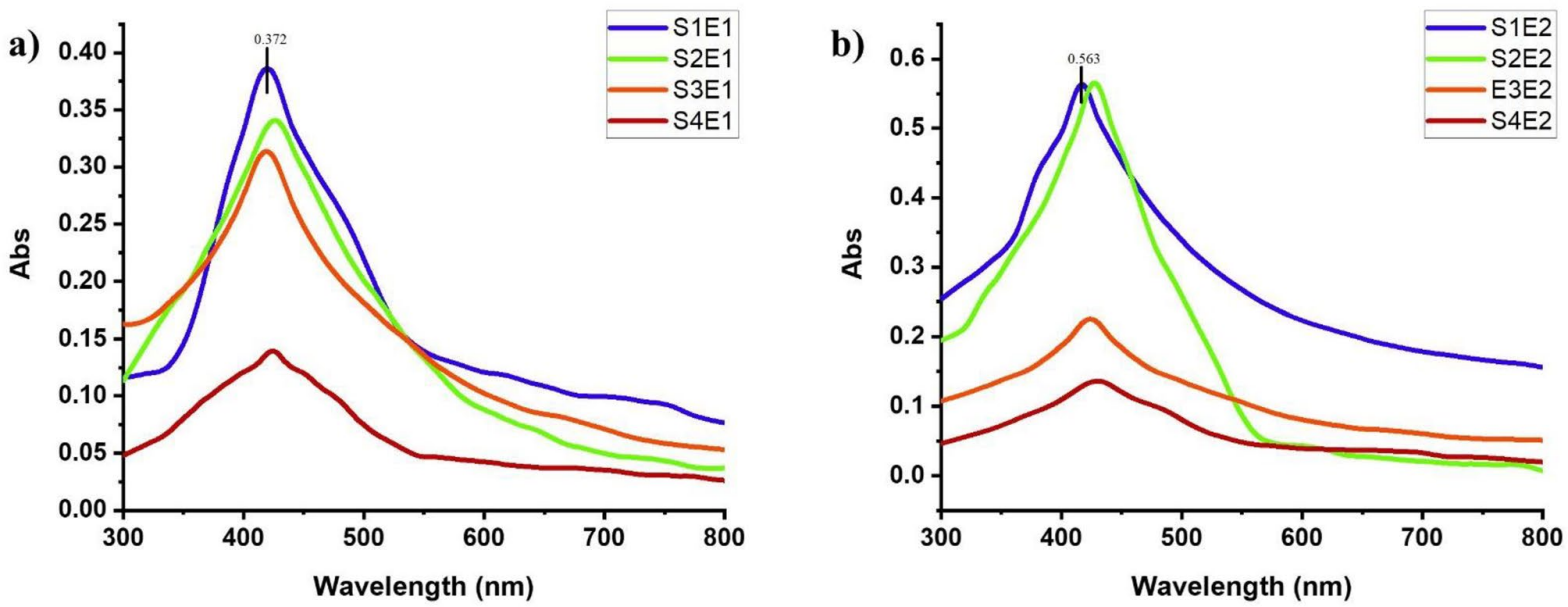
Effect of different solvents on the synthesis of AgNPs using leaf extract of Neem (*Azadirachta indica*) using (**a**) maceration (**b**) boiling method.

The extracts were reacted with silver nitrate solution to synthesize AgNPs at optimized conditions for each solvent. The UV-spectrum of extract S1E1 showed a sharp and clear peak at 426 nm wavelength giving a maximum absorption rate of 0.372 more than S2E1, S3E1, and S4E1. The characteristic peak for S2E1 was shifted to a higher wavelength than other extracts, suggesting the synthesis of larger AgNPs. Among the other four extracts, S1E2, S2E2, S3E2, and S4E2, the S1E2 and S2E2 extracts gave a more absorption rate of 0.563 and 0.566, respectively, than other solutions. Still, the S1E2 extract was selected for further use because the peak of S2E2 was slightly shifted to a higher wavelength suggesting the synthesis of larger-sized NPs [Fig. 3a (maceration method) and Fig. 3b (boiling water method)].

#### Physiochemical characterization of silver nanoparticles

The AgNPs were synthesized using aqueous extracts, S1E1 and S1E2, of leaves powder of Neem plant prepared via maceration and boiling, respectively. The S1E1 extract was reacted in a 1:1 ratio with 2 mM silver nitrate solution, kept at 70 °C, and after 3 h of reaction, the solution was taken in Eppendorf’s tube to purify the NPs. Similarly, the AgNPs synthesized using 20 mL of S2E1 extract of Neem plant reacted with 10 mL of 1 mM silver nitrate soliton at 70 °C for 3 h and were purified and stored. The pH of the solutions for both extracts was maintained at 11. The purified AgNPs using both extracts were subjected to various techniques given below.

**Figure 4:**
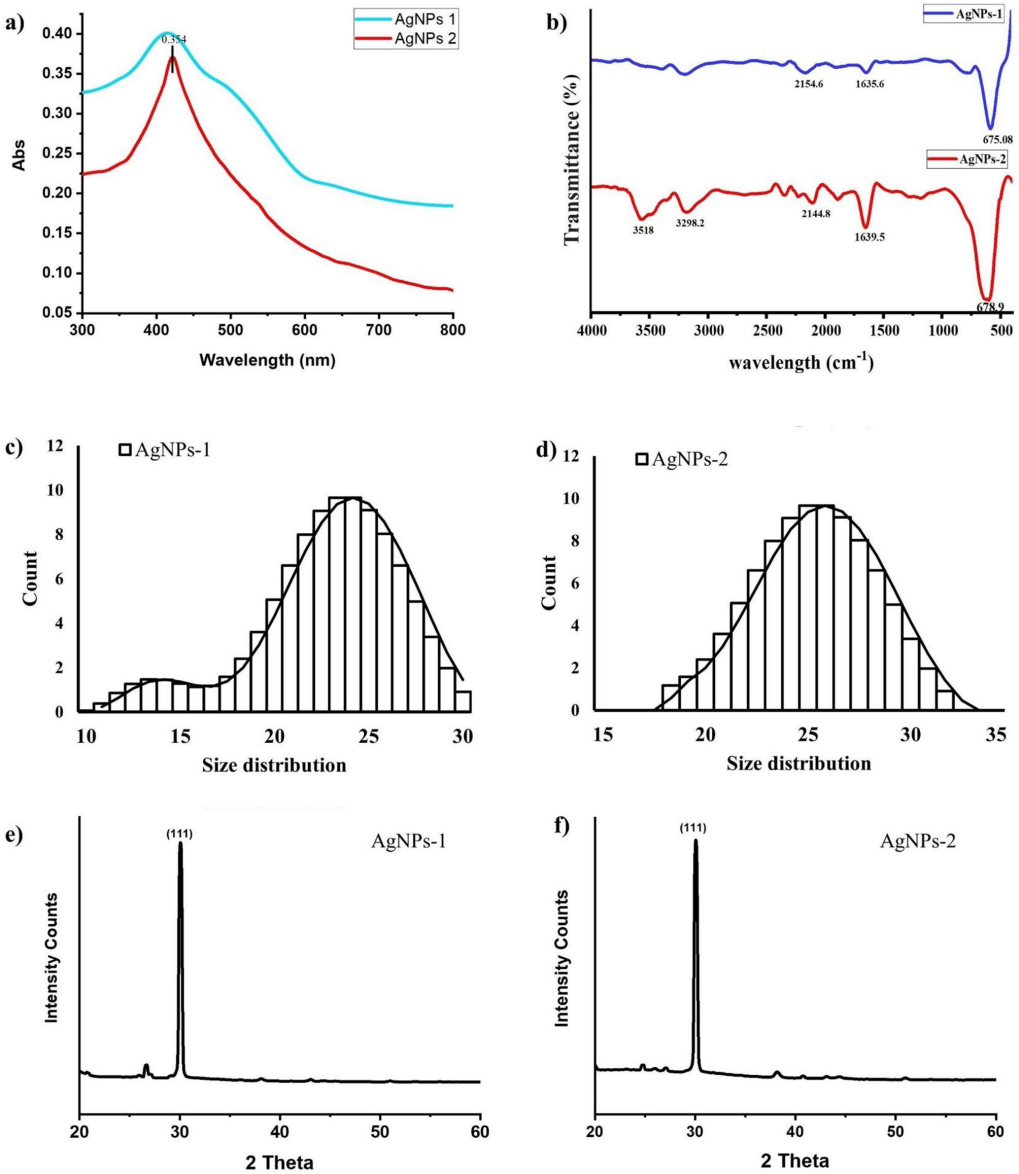
Physiochemical characteristics of green synthesized silver nanoparticles using aqueous extract of Neem plant (**a**) UV spectrum (**b**) FTIR spectrum (**c**) size distribution of sample 1 (**d**) size distribution of sample 2 (**e**) XRD spectrum of sample 1 (**f**) XRD spectrum o of sample 2.

The purified AgNPs of both extracts were dissolved in distilled water and subjected to UV-spectrophotometry within 300–800 nm to measure the absorption spectrum. A clear but broad characteristic peak for AgNPs-1 and a sharp one for AgNPs-2 can be seen in Fig. 4a due to the surface plasmon resonance phenomenon of the nanoparticles. AgNPs-2 exhibited a sharp peak as compared to AgNPs 1.

The particle size of sample AgNPs-1 using the Zeta sizer was recorded as 10–30 nm, shown in Fig. 4c. The maximum number of NPs was between 15 and 30 nm, and the minimum count was between 10 and 15 nm. While the maximum particle size in sample AgNPs-2 was distributed within the range of 22–30 nm, as shown in Fig. 4d.

FTIR analysis was performed to examine the attached functional groups of biomolecules on the surface of AgNPs acting as capping/stabilizing agents. The FTIR spectrum of AgNPs-1 showed absorption bands at 675.08 cm^−1^, 16,335.6 cm^−1^, and 2154.6 cm^−1^, while the FTIR spectrum of AgNPs-2 showed bands at 678.9 cm^−1^, 1639.5 cm^−1^, 2144.8 cm^−1^, 3298.2 cm^−1^ and 3518 cm^−1^ (Fig. 4b). The peaks near 675.08 cm^−1^ and 678.9 cm^−1^ assigned to CH out of plane bending vibrations are substituted ethylene systems –CH=CH (cis). The bands at 1635.6 and 1639.5 cm^−1^ correspond to amide-I arising due to carbonyl stretch (–C=O) in proteins. The peaks at 2154.6 cm^−1^ and 2144.8 cm^−1^ are assigned to the stretching vibration of –C–N of amide I, while the band at 3298.2 cm^−1^ is assigned to C–H (methoxy compounds) and stretching vibration of aromatic compounds and 3518 cm-1 corresponds to –OH stretching vibration indicating the presence of alcohol and phenol involved in capping and stabilizing of AgNPs.

The XRD analysis indicates the synthesis of crystalline nature silver nanoparticles using both extracts (Fig. 4e,f). The size of both samples was determined using the Debye–Scherrer formula. The average size of AgNPs in sample 1 was 32 nm and 24.98 in sample AgNPs-2, determined by the Debye–Scherrer formula close to the values observed in the zeta sizer. A single sharp peak recorded at 2θ degrees of 30.07 for AgNPs-1 and 30.06 for AgNPs-2 is assigned to plane 111 suggesting both samples’ face-centered crystalline structure.

Energy Dispersive Xray (EDX) analysis of both samples, AgNPs-1 and AgNPs-2, shows the peaks in the reign of silver due to its surface plasmon resonance, which is generally at 3 keV, verifying the synthesis of AgNPs. EDX profile of AgNPs-1 showed signals (Fig. 7) for the synthesis of AgNPs along with strong signals for carbon (C) and oxygen (O) as well, which may develop because of phytochemicals attached to the surface of AgNPs acting as capping agents. The profiling of AgNPs-2 suggests the synthesis of AgNPs as the signal can be seen in the region of 3 keV along with signals of other biomolecules (including C, O, Na and Mg) in the sample. However, the signal for AgNPs was stronger in sample AgNPs-2 than in the AgNPs-1. Table 1 presents the elemental composition and concentration of two samples of Siver Nanoparticles (AgNPs), labelled as AgNPs-1 and AgNPs-2.

**Table 1:** Elemental composition and concentration of silver nanoparticles.

Sample	Element	Line type	Apparent concentration	k ratio	Wt%	Wt% sigma	Standard label	Factory standard
AgNPs-1	C	K series	10.93	0.10934	43.21	1.15	C Vit	Yes
	O	K series	23.42	0.0788	44.5	1	SiO_2_	Yes
	Ag	L series	5.88	0.0588	12.29	0.48	Ag	Yes
	Total				100			
AgNPs-2	C	K series	3.23	0.03234	11.41	1.54	C Vit	Yes
	O	K series	10.64	0.03582	24.97	1.41	SiO_2_	Yes
	Na	K series	6.05	0.02554	8.97	0.51	Albite	Yes
	Mg	K series	0.58	0.00384	2.09	0.27	MgO	Yes
	Si	K series	1.52	0.01206	3.38	0.24	SiO_2_	Yes
	Ag	L series	26.61	0.26612	49.18	1.49	Ag	Yes
	Total				100			

**Figure 5:**
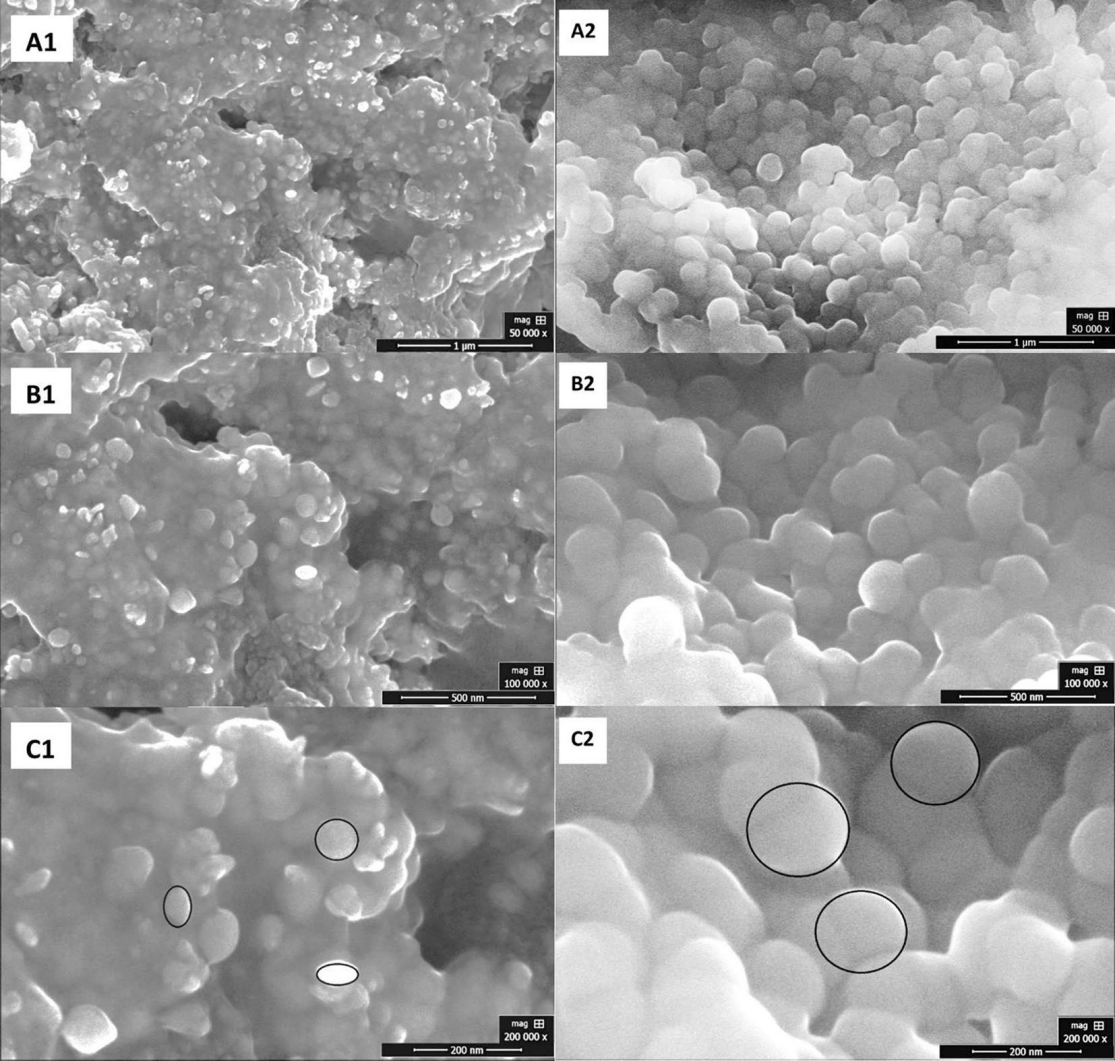
SEM images of green synthesized silver nanoparticles using aqueous extract of Neem plant (**a**) AgNPs-1 and (**b**) AgNPs-2.

The surface morphology of green synthesized silver nanoparticles was studied using a scanning electron microscope (SEM). Smooth spherical to oval shaped AgNPs nanoparticles in aggregated form were synthesized using plant extract S1E1, prepared in distilled water via maceration technique, as shown in Fig. 5a. Silver nanoparticles were spherical with a smooth surface when prepared with plant extract (S1E2) in distilled water using the boiling method (Fig. 5b). The size was estimated to be less than 20 nm in sample AgNPs-1 and less than 30 nm in sample AgNPs-2.

#### Effect of silver nanoparticles on seed germination of tomato

**Figure 6:**
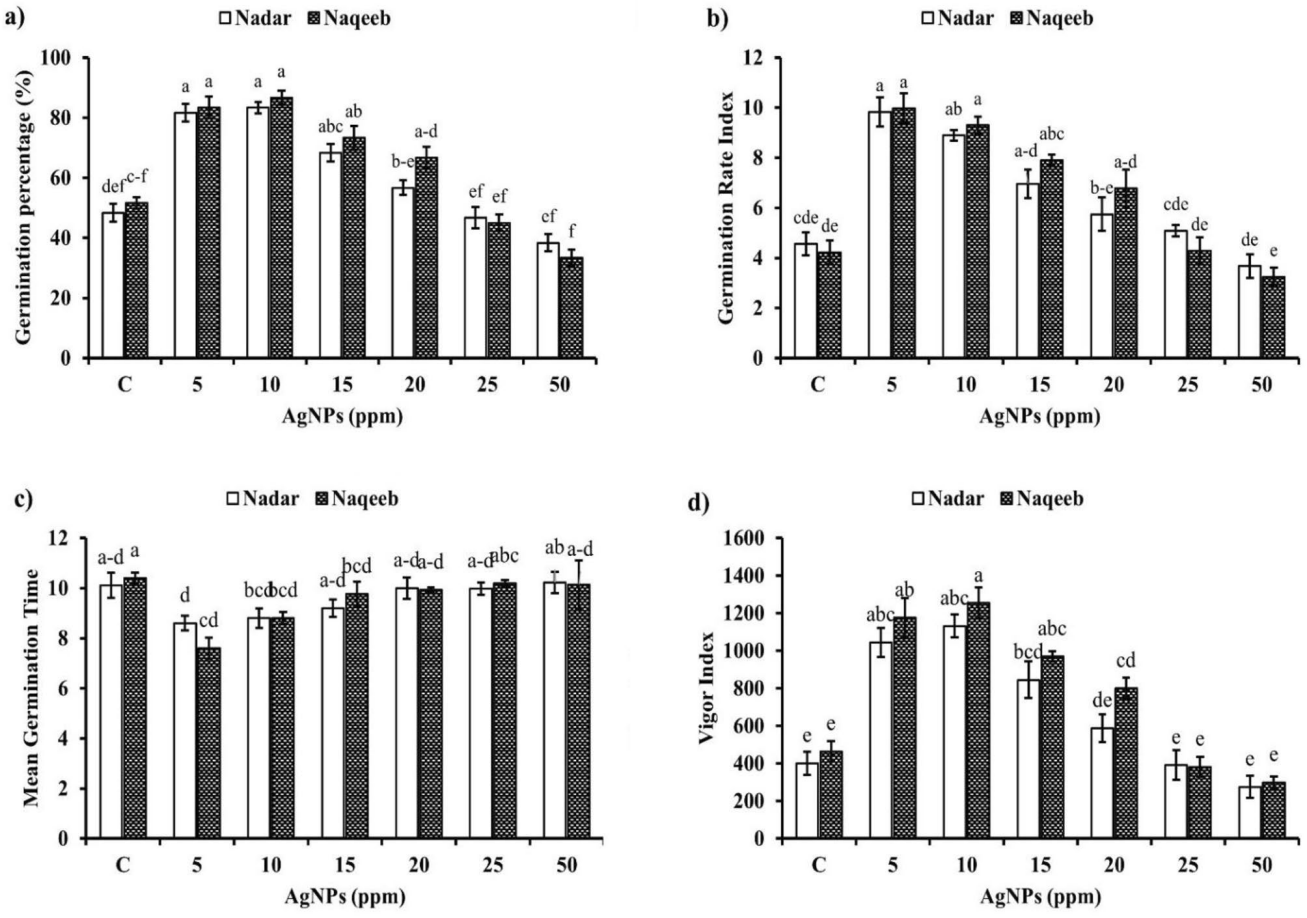
Effect of different concentration of green synthesized silver nanoparticles on (**a**) germination percentage (**b**) germination rate index (**c**) mean germination time and (**d**) vigor index.

This study shows how tomato seeds germinate when exposed to various concentrations (5, 10, 15, 20, 25, and 50 ppm) of green synthesized AgNPs. Silver nanoparticles significantly affected the germination percentage, rate, meantime, and vigor index at 5, 10, 15, and 20 ppm. There was no significant effect on germination time, but the seeds treated with 5 ppm AgNPs took lesser time than other treatments. The maximum germination rate of 83.33% and 86.67 was achieved by Nadar and Naqeeb seeds, respectively, subjected to 10 ppm AgNPs with MGT of 8 days. The germination percentage started to decline as compared to the control when NPs concentration increased up to 25 and 50 ppm. The germination rate index was significantly higher in seeds treated with AgNPs than in control except at 50 ppm suggesting a substantial increase in germination rate due to NPs, as shown in Fig. 6. Silver nanoparticles also improved the seed vigor to a concentration of 20 ppm. The highest seed vigor was recorded by Nadar (1131.50) and Naqeeb (1253.67) at 10 ppm, indicating the optimal concentration (Fig. 6).

#### Effect of silver nanoparticles on growth parameters of tomato plants

**Figure 7:**
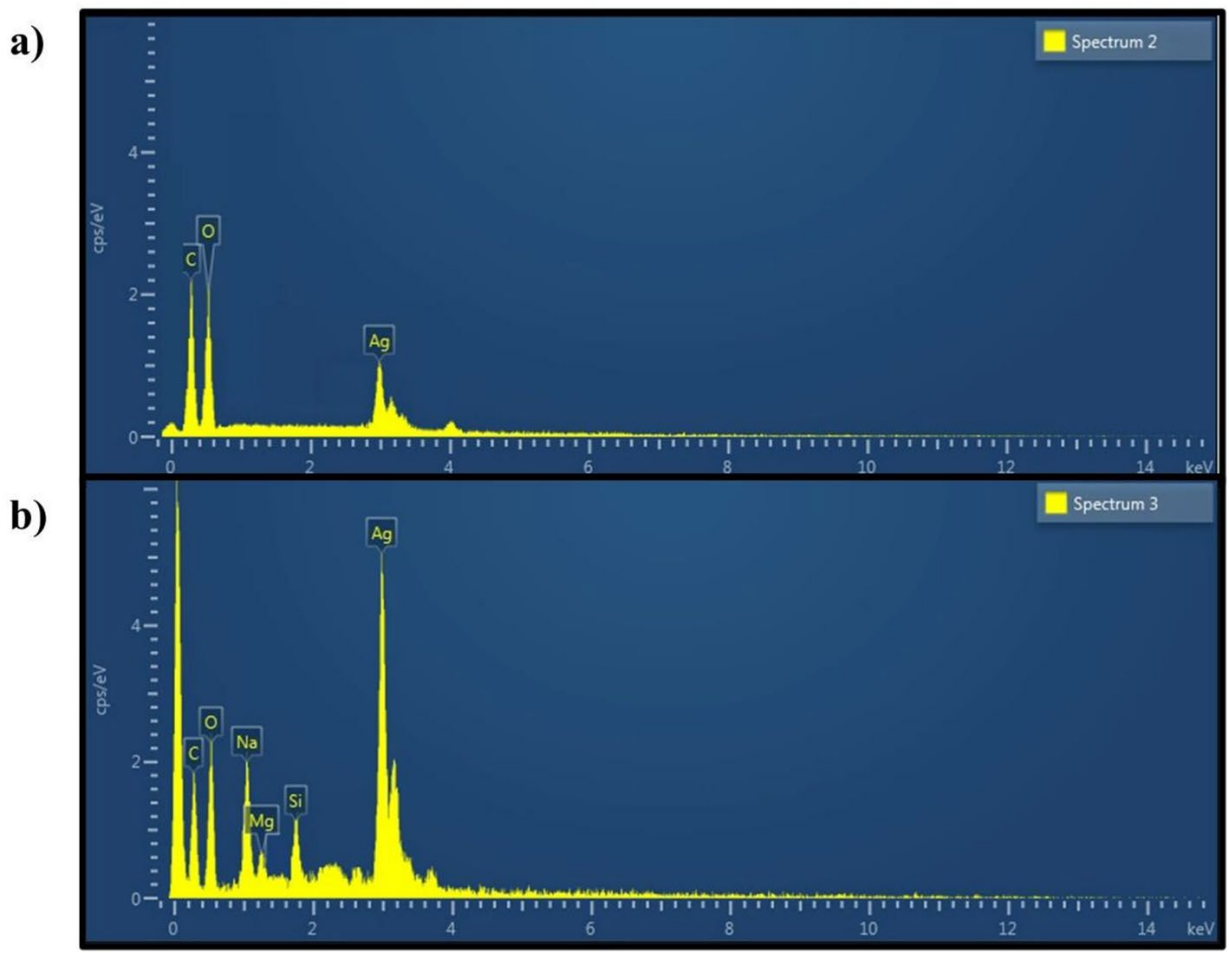
EDX spectrum of green synthesized silver nanoparticles using aqueous extract of Neem plant (**a**) AgNPs-1 and (**b**) AgNPs-2.

Various growth parameters were studied to understand the effects of AgNPs on the growth of Tomato plants. The shoot and root length of Nadar plants was 31.87 cm and 21.40 cm, respectively, in the control group. Plant length increased when treated with 5, 10, 15, and 20 ppm AgNPs. The maximum shoot and root lengths were measured at 10 ppm for the Nadar variety and 5 ppm for the Naqeeb variety. Furthermore, fresh and dry matter of shoot and root in control plants of Nadar varieties were 24.50 g and 9.40 g and 6.79 g, and 3.09 g, respectively, which increased to 44.4 g and 14.82 g and 12.07 g, and 4.67 g when plants were treated with 10 ppm silver nanoparticles. Naqeeb plants followed a similar pattern, as shown in Table 2. Increased AgNPs concentration to 50 ppm reduced fresh and dry matter in both verities (Fig. 7).

**Table 2:** Effect of green synthesized silver nanoparticles on growth parameters of Tomato plants.

Treatments AgNPs (ppm)	Shoot length (cm)		Root length (cm)		Shoot fresh weight (g)		Root fresh weight (g)		Shoot dry weight (g)		Root dry weight (g)	
	Nadar	Naqeeb	Nadar	Naqeeb	Nadar	Naqeeb	Nadar	Naqeeb	Nadar	Naqeeb	Nadar	Naqeeb
Control	31.87^d^ ± 1.228	28.93^d^ ± 1.640	21.40^d^ ± 0.598	15.33^d^ ± 0.652	24.50^e^ ± 2.208	24.67^d^ ± 1.441	9.40^e^ ± 0.708	8.74^d^ ± 1.015	6.79^c^ ± 0.307	6.51^b^ ± 0.448	3.09^c^ ± 0.274	2.40^d^ ± 0.128
5	52.70^a^ ± 1.412	46.95^a^ ± 0.699	30.47^a^ ± 1.235	24.50^a^ ± 0.836	41.07^b^ ± 0.915	42.17^a^ ± 0.927	13.53^b^ ± 0.467	12.63^a^ ± 0.888	11.08^a^ ± 0.661	11.12^a^ ± 0.779	4.26^ab^ ± 0.299	3.55^a^ ± 0.217
10	56.23^a^ ± 1.009	43.23^b^ ± 1.255	31.87^a^ ± 0.457	21.60^ab^ ± 0.940	44.46^a^ ± 0.892	37.40^b^ ± 1.045	14.82^a^ ± 0.598	11.53^b^ ± 0.261	12.07^a^ ± 0.846	10.64^a^ ± 1.091	4.67^a^ ± 0.380	3.27^b^ ± 0.345
15	46.50^b^ ± 0.969	39.10^c^ ± 1.489	26.73^b^ ± 2.235	19.66^bc^ ± 0.522	32.47^c^ ± 1.241	36.72^b^ ± 0.683	12.12^c^ ± 0.275	10.57^c^ ± 0.273	9.04^b^ ± 0.490	10.04^a^ ± 0.701	3.81^abc^ ± 0.333	2.99^c^ ± 0.159
20	41.00^c^ ± 2.395	42.20^b^ ± 0.791	23.40^c^ ± 0.518	17.15^ cd^ ± 0.984	29.47^d^ ± 0.694	37.21^b^ ± 2.295	10.90^ cd^ ± 0.514	10.15^c^ ± 0.352	8.36^b^ ± 0.351	9.69^a^ ± 0.360	3.43^bc^ ± 0.371	2.88^c^ ± 0.182
25	30.30^de^ ± 1.385	29.03^d^ ± 1.228	23.20^ cd^ ± 0.706	16.50^ cd^ ± 2.006	24.27^e^ ± 1.167	27.40^c^ ± 1.352	9.98^de^ ± 0.521	9.13^d^ ± 0.318	6.72^c^ ± 0.392	7.23^b^ ± 0.354	3.16^c^ ± 0.137	2.59^d^ ± 0.176
50	27.23^e^ ± 1.599	28.68^d^ ± 1.442	22.07^ cd^ ± 1.019	16.32^ cd^ ± 1.197	20.93^d^ ± 1.261	25.40^d^ ± 1.405	9.63^de^ ± 1.177	8.79^d^ ± 0.735	5.71^c^ ± 0.331	6.82^b^ ± 0.385	3.15^c^ ± 0.158	2.46^d^ ± 0.220

Treatment mean for each treatment is the average of the three replicates; Mean values with different lower-case alphabets are significantly different at *p* ≤ 0.05 (Duncan’s multiple range test). ± values represent standard error (SE).

Quantification of Photosynthetic Pigments, Alkaloids, Flavonoid, Total Soluble Sugar and Protein Content Photosynthetic pigments are essential parameters, and their amount in leaves reveals the overall health of plants. This study determined the effects of AgNPs concentrations on the Tomato plant’s total chlorophyll and carotenoid content compared to the control group. The total chlorophyll and carotenoid content increased significantly with increasing AgNPs concentrations to 20 ppm. Table 3 suggests an increase in AgNPs to 25 and 50 ppm reduced the amount of chlorophyll and carotenoid restricting the plant growth. Significant effects on alkaloids concentration of silver nanoparticles treated plants could be seen. Plants treated with 5 ppm and 10 ppm produced more alkaloids than control and other concentrations of silver nanoparticles. The total flavonoid concentration in the leaves was also determined. The results revealed an increase in the amount of flavonoids in plants exposed to AgNPs. The total flavonoid content of the Nadar and Naqeeb plants in the control group was 2.548 ± 0.149 and 2.433 ± 0.368 mg QE/g, respectively. The minimum level of total flavonoids in plants of both varieties exposed to 50 ppm silver nanoparticles was 1.936 ± 0.135 and 1.841 ± 0.120 mg QE/g. A similar alkaloid production trend was observed in plants treated with AgNPs.

**Table 3:** Effect of green synthesized silver nanoparticles on physiological parameters of Tomato plants.

Treatments AgNPs (ppm)	Total Chl (mg/g FW)		Carotenoid (mg/g FW)		Alkaloid (mg CE/g)		Flavonoid (mg QE/g)		Soluble Sugar (mg/g)		Protein (mg/g)	
	Nadar	Naqeeb	Nadar	Naqeeb	Nadar	Naqeeb	Nadar	Naqeeb	Nadar	Naqeeb	Nadar	Naqeeb
Control	1.87^e^ ± 0.059	2.41^e^ ± 0.029	0.42^d^ ± 0.054	0.84^e^ ± 0.089	0.176^c^ ± 0.042	0.206^d^ ± 0.044	2.548^d^ ± 0.149	2.433^bc^ ± 0.368	0.406^c^ ± 0.109	0.433^b^ ± 0.104	2.040^d^ ± 0.203	2.048^c^ ± 0.183
5	2.82^b^ ± 0.069	3.79^a^ ± 0.046	0.65^a^ ± 0.039	1.39^a^ ± 0.091	0.260^a^ ± 0.038	0.345^a^ ± 0.059	3.692^b^ ± 0.408	3.867^a^ ± 0.419	0.604^a^ ± 0.059	0.684^a^ ± 0.082	2.964^b^ ± 0.376	3.228^a^ ± 0.210
10	3.11^a^ ± 0.066	3.49^b^ ± 0.091	0.67^a^ ± 0.013	1.29^b^ ± 0.076	0.267^a^ ± 0.057	0.303^b^ ± 0.050	3.995^a^ ± 0.337	3.516^a^ ± 0.316	0.635^a^ ± 0.061	0.644^a^ ± 0.156	3.154^a^ ± 0.122	2.951^b^ ± 0.549
15	2.54^c^ ± 0.020	3.26^c^ ± 0.068	0.60^b^ ± 0.040	1.19^c^ ± 0.022	0.207^b^ ± 0.034	0.273^c^ ± 0.031	3.122^c^ ± 0.181	2.808^b^ ± 0.382	0.541^b^ ± 0.053	0.475^b^ ± 0.119	2.407^c^ ± 0.343	2.239^c^ ± 0.145
20	2.40^d^ ± 0.030	2.93^d^ ± 0.044	0.49^c^ ± 0.031	0.93^d^ ± 0.050	0.161^d^ ± 0.025	0.197^de^ ± 0.028	2.181^e^ ± 0.190	2.333^c^ ± 0.289	0.388^c^ ± 0.105	0.427^b^ ± 0.055	1.884^d^ ± 0.318	1.952^ cd^ ± 0.236
25	1.74f. ± 0.029	2.13f. ± 0.095	0.34^e^ ± 0.017	0.78f. ± 0.088	0.137^e^ ± 0.035	0.172^ef^ ± 0.036	1.973f. ± 0.152	2.144^ cd^ ± 0.301	0.345^d^ ± 0.034	0.370^c^ ± 0.147	1.695^e^ ± 0.239	1.750^de^ ± 0.177
50	1.44^ g^ ± 0.069	1.89^ g^ ± 0.066	0.31f. ± 0.031	0.63^ g^ ± 0.020	0.123^e^ ± 0.049	0.153f. ± 0.034	1.936f. ± 0.135	1.841^d^ ± 0.120	0.323^d^ ± 0.056	0.301^d^ ± 0.142	1.422f. ± 0.148	1.529^e^ ± 0.228

Treatment mean for each treatment is the average of the three replicates; Mean values with different lower-case alphabets are significantly different at *p* ≤ 0.05 (Duncan’s multiple range test). ± values represent standard error (SE).

Silver nanoparticles also greatly affected the protein content accumulation in both varieties of Tomato plants. The protein content started increasing in plants treated with AgNPs at 20 ppm. Further increase in the concentration of AgNPs reduced the protein amount. The maximum protein content of 3.154 ± 0.122 in 10 ppm treated Nadar plants and 2.951 ± 0.549 in Naqeeb plants was found. Table 3 shows the considerable change in soluble sugar as well. The sugar content was increased from 0.406 ± 0.109 (control) and 0.433 ± 0.104 to 0.635 ± 0.061 (Nadar), and 0.644 ± 0.156 at 10 ppm in AgNPs treated plants.

## Discussion

Nanomaterials have gained a lot of interest and application in various fields. Scientists have used traditional synthesis methods of nanomaterials like the physical, chemical, thermal, and hydrothermal that are extremely expensive, hazardous, and use toxic chemicals^45–47^. Therefore, the green synthesis of nanoparticles is found to be a more advanced, widespread, and remarkable area of nanotechnology. Using plants to synthesize nanoparticles is easy, single-step, nonpathogenic, cost-effective, non-toxic, and sustainable as it uses renewable resources and is environment friendly^9,48^. Various physical factors control the silver nanoparticles’ size, shape, and stability during green synthesis^49^. Silver nanoparticles acquire unique optical properties due to surface plasmon resonance (SPR) generated from free electron movement. Therefore, interacts with a specific wavelength of visible light reported between 400 and 500 nm and develops a brown color in the solution depicting the synthesis of silver nanoparticles^50,51^.

The present work synthesized silver nanoparticles using Neem plant leaves extract and optimized the factors affecting the green synthesis protocol. Temperature affects the size and shape of silver nanoparticles and the rate at which silver nanoparticles are synthesized^52^. It is suggested that low temperature delays the synthesis of silver nanoparticles. Silver nanoparticles start synthesizing at 40 °C, while better and faster development of silver nanoparticles requires an elevated temperature of about 60–80 °C^53^. Smaller-sized silver nanoparticles are synthesized because of higher temperatures in a short time, and the UV spectrum of such nanoparticles moves to a lower wavelength (blue shift). In contrast, a broad peak at a higher wavelength (red shift) is attributed to agglomeration or an increase in the size of the particles at low temperatures^54^. Kredy et al.^55^ showed that lower reaction temperatures resulted in larger nanoparticles, whereas high temperatures produced small nanoparticles. Furthermore, it was found that *Vitex agnus-castus* leaf extract could lead to a fast reduction of Ag+ ions, even at a low temperature of 40 °C. In contrast, the efficient production of AgNPs was observed in another study when the temperature was between 60 and 80 °C^53^. The AgNPs of 17 nm were synthesized using *Ocimum sanctum* (Tulsi) leaf extract, the yield of production was increased after 15 min and continued to increase with increasing the time up to 3 h^56^.

The reduction of silver nanoparticles starts after adding silver nitrate to the plant extract, as evidenced by the color change from light green to brown^57^. After an optimum time for the formation of stable silver nanoparticles, silver nanoparticles start to agglomerate, resulting in larger particle sizes^58^. The formation of silver nanoparticles is also concerned with the optimum amount of metal precursors and the reducing agents in the plant extract. The availability of metal ions in the reaction mixture influences the yield of nanoparticles. The higher the metal precursor concentration and the reducing agent, the greater the possibility of obtaining AgNPs^59^. According to previous studies, pH is another important parameter affecting nanoparticle synthesis. The higher pH increases the availability of biochemicals for reducing and capping silver nanoparticles, producing highly stable and smaller-sized silver nanoparticles. The acidic pH prevents the formation of silver nanoparticles and contributes to particle instability^23,54^.

FTIR analysis makes us understand the role of biomolecules in plant leaf extract for reducing silver to silver nanoparticles and their stability in acting as capping agents^60^. The FTIR analysis reveals the involvement of alkanes, alkynes, amines, carboxylic groups^61^, methylene^62^, and various other biomolecules in reducing and stabilizing green synthesized silver nanoparticles^63^. Biomolecules extracted in different solvents make changes in size, shape, stability, and peaks of FTIR spectra, demonstrating the significance of the extraction method and solvent type^64^. XRD pattern and SEM analysis of AgNPs synthesized by Neem and other plants suggested the synthesis of crystalline structure spherical silver nanoparticles^65–67^.

Green synthesized nanoparticles have tremendous biological applications as antibacterial, antifungal, and anti-cancerous agents in medicine and agriculture^68,69^. The silver nanoparticles promote crop yield with better plant growth even at low concentrations^70^. Metallic nanoparticles improve the efficiency of photosynthetic systems in plants, increasing the content of photosynthetic pigments, which improves plants’ growth and weight. Foliar application of silver nanoparticles on Fenugreek plants gradually increased all growth parameters compared to untreated plants using 40 mg/L and decreased at 60 mg/L but still higher than the control^71^. Silver nanoparticles (9–35 nm size) synthesized using leaves of *Ocimum basilicum* L., and *Mangifera indica* L. increased the shoot length, fresh and dry biomass, chlorophyll, carbohydrates, and protein content of wheat plants at 20 and 40 ppm. Inhibitory effects were also observed beyond these concentrations^72^.

## Conclusions

Nanotechnology plays a vital role in introducing tools for disease management, enhancing disease diagnostics, and developing new measures. Green synthesis uses simple procedures, readily available raw materials, environment-friendly, cost-effective, and easily scaled up for large-scale synthesis of nanoparticles. Furthermore, there is no need to use high temperatures, pressure energy, and toxic chemicals. The optimization of different factors resulted in the formation of stable nanoparticles and gave maximum yield making sure to utilize the resources well. The silver nanoparticles improved the germination rate and plant growth of Tomato plants, enhancing the production of chlorophyll, carotenoids, alkaloids, and flavonoids. So, green synthesized silver nanoparticles have a noticeable effect on the agricultural world in minimizing environmental contamination and promoting plant growth. However, the physicochemical characteristics of the generated AgNPs may exhibit specificity towards a particular crop species and the prevailing climatic conditions of a particular locality. Therefore, further research is required to assess the antimicrobial efficacy of silver nanoparticles (AgNPs) in practical agricultural settings. Additional investigation should be focused on determining the optimal threshold concentrations of silver nanoparticles (AgNPs) that effectively reduce the occurrence or severity of diseases in plants, while avoiding any negative effects on non-target organisms and soil fertility.

## Methods

### Plant material, preparation of leaf extract and reagent

*Azadirachta indica* (Neem plant) leaves were collected from the botanical garden, University of the Punjab, Lahore, and washed using tap water several times to remove all the dust. Then, leaves were dried at room temperature under shade, ground with the help of an electric grinder, and stored. Seeds of Tomato varieties were brought from Ayyub Agriculture Research Institute, Faisalabad. All the required chemicals, i.e., silver nitrate (AgNO_3_), methanol, dimethyl sulfoxide (DMSO), dichloromethane (CH_2_Cl_2_), and potassium bromide (KBr), acetone, *n*-hexane, acetic acid, sulphuric acid, and phosphate buffer were bought from Sigma-Aldrich through Science Traders.

The plant extracts were prepared using maceration and boiling methods. Firstly, powdered material of Neem leaves was added to each solvent with a 1:10 ratio, respectively. The mixtures were kept overnight at room temperature, followed by filtration through Whatman’s No. 1 filter paper, and the filtrate was used to synthesize silver nanoparticles. Then using the same ratio of leaves powder in mentioned solvents, the mixtures were boiled for 30 min, cooled down, and filtered with Whatman’s no. 1 filter papers. The extracts were labeled as S1E1, S2E1, S3E1, S4E1, S1E2, S2E2, S3E2, and S4E2 representing the solvent type (distilled water S1, methanol S2, DMSO S3, and DCM S4) and extraction method (maceration E1and boiling method E2) respectively. Molar solutions of silver nitrate, i.e., 1, 2, 3, and 4 mM, were prepared by mixing the required amount of silver nitrate in distilled water.

### Green synthesis of silver nanoparticles

#### Effect of temperature, time, pH, reagent concentration and plant extract volume

The temperature of the reaction mixture was optimized by adding 10 mL of freshly prepared extracts (mentioned in Sect. 2.2) with 10 mL of 1 mM AgNO3 solution in glass vials. An incubator was used to set up the experiment for an hour to check the effect of various temperatures (30 °C, 40 °C, 50 °C, 60 °C, 70 °C, and RT room temperature). Firstly, the synthesis of AgNPs was indicated by the reaction mixture’s color change due to the reduction of Ag+ to Ag0. Then each solution was taken in Eppendorf’s tube and centrifuged. The pellet was dissolved in distilled water and centrifuged again. The centrifugation process was repeated several times to purify the nanoparticles. Each solution was subjected to UV-spectrophotometer (Shimadzu UV-1800) within the 300 nm to 800 nm wavelength to observe the spectrum. The temperature producing maximum AgNPs was chosen for the further optimization procedure.

The incubation time was optimized using 10 mL of each freshly prepared leaf extract with 10 mL of 1 mM AgNO3 solution in glass vials. The solutions were incubated in the dark at the selected temperature for 1, 2, 3, 4, 5, and 6 h. The change in color of the reaction mixture indicated the synthesis of AgNPs. The color change in the reaction mixture was observed. Purified silver nanoparticles were subjected to UV-spectroscopy for further confirmation of synthesis after each time interval. The incubation time showing the maximum production of AgNPs was used for the further optimization procedure.

**Figure 8:**
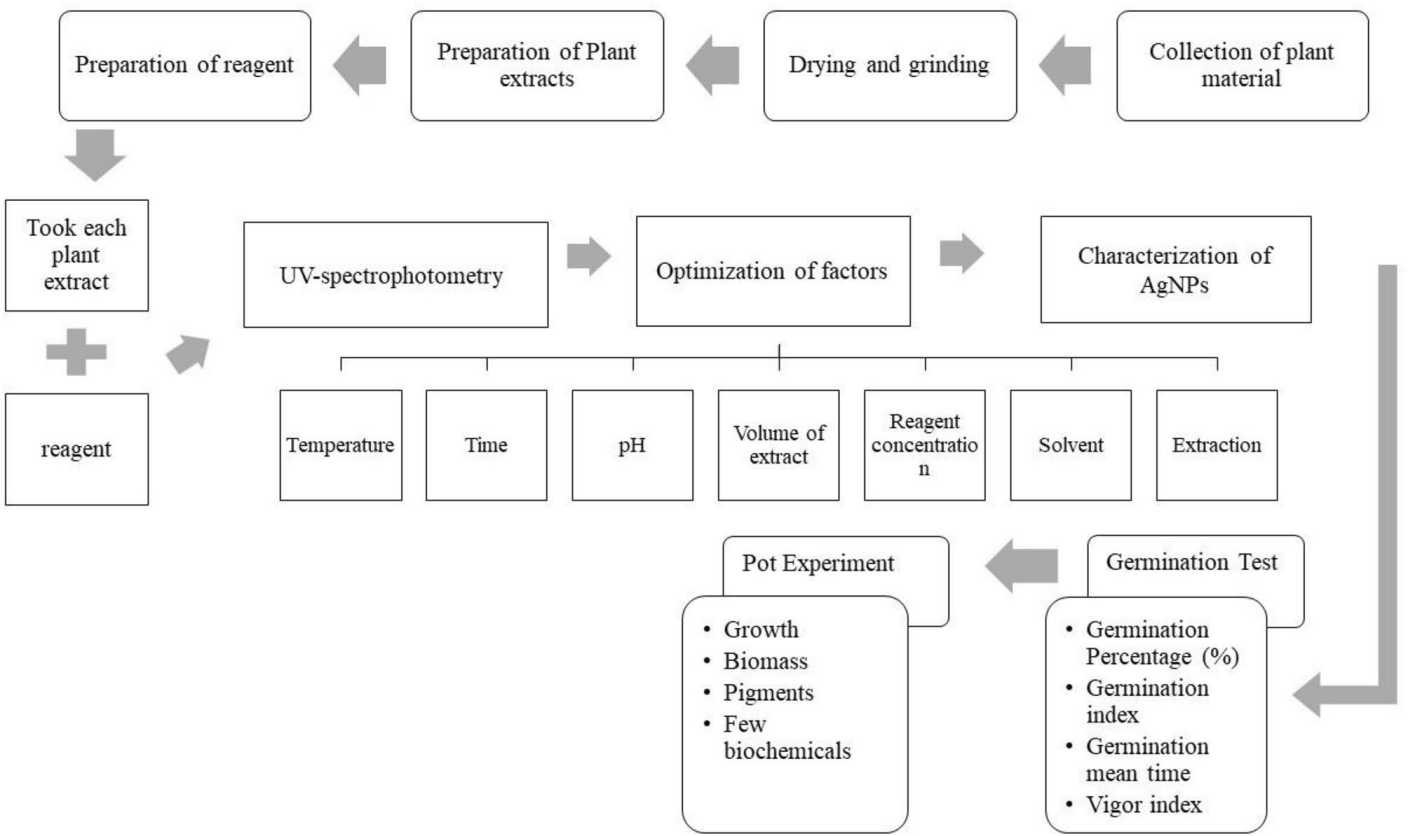
Schematic diagram of methodologylogy.

Various pH values were considered to find the optimum value for synthesizing silver nanoparticles using the Neem plant’s dried leaves. The freshly prepared leaf extract was added to 1 mM silver nitrate solution in a respective vial. The pH of the solution was maintained at 5 and kept in an incubator at the selected temperature. The color change was observed at first for the synthesis of AgNPs, and then the solution was subjected to UV-spectrophotometry after an optimized time. The process was repeated at pH 7, 9, 11, and 13. NaOH and HCl were used to maintain the pH of the solutions. The pH at which the maximum yield of nanoparticles was given was selected for further procedures (Fig. 8).

Various concentrations of silver nitrate, i.e., 1 mM, 2 mM, 3 mM, and 4 mM were used to optimize the reagent concentration for the synthesis of AgNPs. Prepared plant extracts were reacted with different concentrations of AgNO3 in a 1:1 ratio at a selected temperature and time. After observing the color change, the resulting solutions were subjected to UV–vis spectroscopy to produce a spectrum and monitored. The concentration of silver nitrate solution producing the maximum AgNPs was selected for further procedure.

Optimization of used plant extract volume was performed using different amounts of plant extract, i.e., 10 mL, 20 mL, 30 mL, 40 mL, and 50 mL treated with 10 mL of AgNO_3_ solution. Color change of the solutions was observed after reaction completion to confirm the synthesis of silver nanoparticles. The synthesized AgNPs were centrifuged several times until AgNPs were purified and assessed using a UV–vis spectrophotometer within 300–800 nm. The volume of Neem leaf extract producing maximum AgNPs was selected for further procedure^73–77^.

#### Effect of solvent type

The prepared plant extracts using two different methods were used to synthesize AgNPs at already optimized conditions to select the maximum NPs producing solvent for each technique. The resulting solutions were subjected to UV–vis spectroscopy to monitor the maximum production of AgNPs. The prepared plant extracts using two different methods were used to synthesize AgNPs at already optimized conditions to select the maximum NPs producing solvent for each technique. The resulting solutions were subjected to UV–vis spectroscopy to monitor the maximum production of AgNPs.

#### Characterization of green synthesized silver nanoparticle

Green synthesized silver nanoparticles at optimized conditions were subjected to the following techniques to determine their size, surface structure, morphology, and other characteristics.

#### UV-spectrophotometric analysis

This technique was used to determine the formation of silver nanoparticles. The purified silver nanoparticles were dissolved in 5 mL of distilled water using a vortex meter and subjected to UV- vis analysis. The absorption spectrum within the wavelength range of 300–800 nm was taken on a UV–vis spectrophotometer (Shimadzu UV-1800)^78^.

#### Fourier transform infrared (FTIR) and particle size analysis

Fourier transform infrared spectroscopy (FTIR) was used to study the organic functional groups attached to the surface of AgNPs. The synthesized nanoparticles were purified and dried at 60 °C. The dried samples were mixed with a fine powder of potassium bromide (KBr) and analyzed by FTIR (Bruker Alpha Platinum ATR). The size surface charge of silver nanoparticles was determined using a zeta sizer. Particle size measurement was based on the time-dependent fluctuation of laser light scattering by the nanoparticles undergoing Brownian motion^78^.

#### X-ray diffraction and energy dispersive X-ray (EDX) analysis

X-ray diffraction (XRD) was used to examine the overall oxidation state and crystal structure of AgNPs. The energy-dispersive X-ray technique was used to study the elemental composition of synthesized AgNPs^78^.

#### Scanning electron microscopic analysis

Scanning electron microscopy (SEM) was performed to visualize synthesized particles’ morphology at submicron scale and elemental information at the micron scale (FEI Nova 450 NanoSEM). Thin films of the sample were prepared on a carbon-coated copper grid. A drop of AgNPs was placed on carbon-coated copper grids and allowed to stand for two min, and the excess solution was removed using a blotting paper, and then the film on the grid was allowed to dry at room temperature and exposed to an electron beam^79^.

#### Effect of silver nanoparticles on seed germination of tomato

The effect of green synthesized AgNPs on seed germination of the Tomato was determined in Petri plates. Seeds were exposed to 5% sodium hypochlorite solution for 2–3 min, repeatedly washed with distilled water, and soaked in prepared concentrations (5, 10, 15, 20, 25, and 50 ppm) of silver nanoparticles for approximately 2 h. Seeds were transferred to sterilized Petri plates and placed on three layers of filter paper with 1 cm distance between them. The Petri plates were covered, sealed with a tap, and incubated at room temperature for two weeks. The seed germination rate, mean germination time, and percentage were measured using the formulas below.

$${\text{Germination }}\,{\text{percentage}} = \frac{{{\text{Number }}\,{\text{of }}\,{\text{seeds }}\,{\text{germinated}}}}{{{\text{Total}}\,{\text{ number }}\,{\text{of }}\,{\text{seeds}}}} \times 100$$

$${\text{Germination}}\,{\text{ Rate }}\,{\text{Index }}\left( {{\text{GRI}}} \right) = \frac{{{\text{G}}1}}{1} + \frac{{{\text{G}}2}}{2} + \cdots + \frac{{{\text{Gx}}}}{{\text{x}}}$$

Calculations of the germination rate index show the percentage of germination per day, so the higher the percentage and the shorter the duration, the higher the GRI.

$${\text{Mean }}\,{\text{Germination }}\,{\text{Time }}\left( {{\text{MGT}}} \right) = {\Sigma }\frac{{{\text{NiDi}}}}{{\text{n}}}$$

Ni is the number of germinated seeds until the ith day, Di is the number of days from the experiment until the ith counting, and n is the total number of germinated seeds.

$${\text{Seed}}\, {\text{vigor}} = {\text{Seedling }}\,{\text{length }}\left( {{\text{cm}}} \right) \times {\text{Germination }}\,{\text{percentage}}$$

### Effect of silver nanoparticles on tomato under greenhouse conditions

#### Experimental design and application of silver nanoparticles

A pot experiment was performed to determine the effect of green synthesized AgNPs on the growth and physiological response of Tomato plants during the season of 2020–2021. Seeds of two Tomato varieties, Nadar and Naqeeb, were brought from the Vegetable Research Institute of Ayyub Agricultural Research Institute (AARI), Faisalabad, and used to prepare seedlings. The cleaned clay pots filled with loamy soil were placed in a wire-enclosed area and arranged according to a particular variety, respective treatment, and replicate number following a randomized complete block design (RCBD). Already prepared healthy seedlings were transplanted into pots and kept one plant per pot. Standard agricultural practices were used to irrigate and maintain the plants. Solutions of silver nanoparticles in various concentrations, i.e., 5, 10, 15, 20, 25, and 50 ppm, were applied on plants using the foliar spray until run-off at 15 days intervals. Tap water was given to plants taken as control.

#### Plant growth analysis

Growth parameters, including shoot length, root length, and fresh weight of the plants, were recorded after 55 days of transplantation. The plants were removed carefully from the pots, packed in labeled bags, and brought to the laboratory. Plants were washed with tap water and blotted to remove excess moisture. Then, plants were dried in a hot air-drying oven at 70 °C for 72 h and measured the weight.

#### Quantification of photosynthetic pigments, alkaloids, flavonoid, total soluble sugar and protein content

Plant leaves were cut into 0.5 cm segments, and their extraction was made with 80% acetone overnight at − 10 °C. Then, for five minutes, the extract was centrifuged at 14000×*g*, and by using a spectrophotometer, the absorbance of the supernatant was recorded at 480, 645, 663 nm, respectively^80,81^. The total chlorophyll and carotenoids were measured by using the following formula.

Tomato leaves of treated plants were cut into 0.5 cm pieces, extracted in 80% acetone, and centrifuged at 14000×*g*. The supernatant was used to take the absorbance in a spectrophotometer at Δ480, Δ645, and Δ663 nm^80,81^. The extract prepared in acetone was used again to calculate carotenoid content^82^. The formulas below were used to calculate chlorophyll and carotenoids.

$$\begin{aligned} & Total{ }\,Chlorophyll{ }\left( {\frac{mg}{g}FW} \right) = \left( {0.0202{ } \times OD{ }645} \right) + { }(0.0082{ } \times OD{ }663 \\ & {\text{Carotenoids }}\left( {{\text{mg}}/{\text{g FW}}} \right) \, = \, ({1}000 \, \,{\text{OD }}\,{48}0 \, - { 3}.{27 }\left( {Chl{\text{a}}} \right) \, {-}{ 1}0{4 }\left( {Chl{\text{b}}} \right)/{227} \\ \end{aligned}$$

Alkaloids were extracted from 2 g samples of the dried plant organs with n-hexane (3 × 20 ml) followed by MeOH (3 × 20 ml). The extract was kept at room temperature, and each cycle with hexane lasted for 24 h. The extract was evaporated in a rotary film evaporator (RFE). As the last step, each tube was rinsed with 15 mL of distilled water, which was added to the flask. The non-alkaloids in the mixture were removed with CH_2_Cl_2_ (3 × 1/3 of the total volume). The acidic aqueous solution was made alkaline (pH 8 to 10) by adding 10% NaOH, and the contents were extracted with CH_2_C1_2_ to obtain the alkaloids^83^. At this stage, the aqueous phase was discarded, and the organic phase was dried in RFE. The residue was dissolved in 1 mL CH_2_C1_2_ and stored until further use.

The total flavonoid production in the leaves of Tomato plants was determined based on a method of^84^. Firstly, 1 g piece of leaf was ground with 1% acetic acid ethanol solution in 1:99 and centrifuged at 12,000xg for 15 min. The supernatant was used for further procedure and transferred into a polypropylene tube. The tube was incubated for 10 min at 80 °C in a water bath and then cooled to room temperature. The absorption of the obtained supernatant was observed at Δ270, Δ300, and Δ330 nm in a spectrophotometer. Total flavonoid content was calculated using the extinction coefficient of 0.033 mM/cm.

The method of Nelson^85^ was used to determine the total soluble sugar. For that purpose, 0.5 g of fresh leaf from the Tomato plant was taken in 10 mL of ethanol, incubated for 60 min at 60 °C, cooled down, and then filtered. The extract was mixed with 1 mL of phenol and 5 mL of H_2_SO_4_. The absorbance was recorded at Δ485 nm in a spectrophotometer, and 80% ethanol was taken as blank.

The protein content in the leaves of treated plants was measured using a method by Lowry^86^. The leaf of 0.1 g was added to the phosphate buffer and crushed in a pre-chilled pestle and mortar. The mixture was then centrifuged at 3000×*g* for 10 min. The 0.1 mL supernatant was taken in a test tube, and dH_2_O was added to make it up to 1 mL. The 0.5 mL of the resulting solution was mixed with 5 mL of D reagent, and after 10 min of incubation, 0.5 mL reagent E was mixed vigorously using a vortex meter. The absorption of the mixture and blank was taken at Δ600 nm. Using the BSA standard curve, the protein content of each tested sample was calculated.

## Research statement involving plants

The leaves of neem plants were collected from botanical garden with permission taken from in charge botanical garden, University of the Punjab, Lahore. Seeds of tomato varieties were brought from vegetable research institute, Ayyub Agriculture Research Institute, Faisalabad with permission taken from Director.

## Data analysis

All the datasets were subjected to ANOVA, and the treatment means were analyzed by *using* computer-aided software SPSS (Version 20) at *LSD* < 0.05 whereas the means related to the absorption and wavelength values of AgNPs were plotted in graphs using Origin Pro 2021b.

## Data Availability

The datasets used in the current study are available from the corresponding author on reasonable request.
